# Exogenous brassinosteroids promotes root growth, enhances stress tolerance, and increases yield in maize

**DOI:** 10.1080/15592324.2022.2095139

**Published:** 2022-07-01

**Authors:** Hao Zhang, Dan Zhao, Ziyan Tang, Ying Zhang, Ke Zhang, Jingao Dong, Fengru Wang

**Affiliations:** a State Key Laboratory of North China Crop Improvement and Regulation, Key Laboratory of Hebei Province for Plant Physiology and Molecular Pathology, College of Life Sciences, Hebei Agricultural University, Baoding, Hebei, China; b College of Life Sciences, Hengshui University, Hengshui, Hebei, China; c Pear Engineering and Technology Research Center of Hebei, College of Horticulture, Hebei Agricultural University, Baoding, Hebei, China; d College of Plant Protection, Hebei Agricultural University, Baoding, Hebei, China; e Ministry of Education Key Laboratory of Molecular and Cellular Biology, Hebei Collaboration Innovation Center for Cell Signaling, Hebei Key Laboratory of Molecular and Cellular Biology, College of Life Sciences, Hebei Normal University, Shijiazhuang, Hebei, China

**Keywords:** Brassinosteroids, maize, yield, resistance

## Abstract

Brassinosteroids (BRs) regulate of maize (*Zea mays* L.) growth, but the underlying molecular mechanism remains unclear. In this study, we used a multi-disciplinary approach to determine how BRs regulate maize morphology and physiology during development. Treatment with the BRs promoted primary root the elongation and growth during germination, and the early development of lateral roots. BRs treatment during the middle growth stage increased the levels of various stress resistance factors, and enhanced resistance to lodging, likely by protecting the plant against stem rot and sheath rot. BRs had no significant effect on plant height during late growth, but it increased leaf angle and photosynthetic efficiency, as well as yield and quality traits. Our findings increase our understanding of the regulatory effects of BR on maize root growth and development and the mechanism by which BR improves disease resistance, which could further the potential for using BR to improve maize yield.

## Introduction

Maize (*Zea mays* L.) is the third largest food crop in China. It is also an important source of human food, animal feed and industrial raw materials. The planting area and output of corn in China rank first in the world. Corn plays an important role in China’s agricultural production and national economy.^1^,^2^

Biotic and abiotic stresses affect maize yields worldwide and improving stress tolerance provides an important approach for improving crop yields. One hallmark of stress conditions is high levels of reactive oxygen species (ROS) such as superoxide and hydrogen peroxide (H_2_O_2_). These ROS typically cause membrane lipid peroxidation under stress conditions^3,4^ such as high temperature, high salt, alkali, and strong light, and the content of malondialdehyde (MDA) reflects the degree of lipid peroxidation of plant cell membranes. High MDA content indicates a high degree of lipid peroxidation and suggests that the cell membrane is seriously damaged.^5–7^

Plants express various enzymes that help them detoxify ROS. Superoxide dismutase (SOD)^8^ effectively enhances the stress tolerance of plants by catalyzing the disproportionation of superoxide anion free radicals, thus limiting damage by ROS to the cell membrane system.^9^ Peroxidase (POD) is an important protective enzyme for preventing oxygen free radical damage in plants, which is closely related to stress tolerance. H_2_O_2_ is an oxidative metabolite in cells. Higher contents of H_2_O_2_ cause greater damage to cell sand reduce the ability of plants to tolerate stress.^10–12^ Ascorbate peroxidase (APX2) also scavenges hydrogen peroxide in plant cells.^13,14^

Plants also produce various small molecules to resist stress. For example, the content of proline in plants reflects the stress tolerance of plants to a certain extent. Stress conditions cause the accumulation of proline in plants, which is dependent on changes in the activity of 1-pyrroline-5-carboxylic acid synthase (*P5CS*)^15,16^ a key enzyme in proline biosynthesis. In addition, the higher the soluble sugar content of plants, the smaller the possibility of cell water loss, increasing the possibility of plant survival under stress and therefore improving stress tolerance.^17,18^

The plant root system functions in absorbing water and nutrients from the soil, storing photosynthetic products, fixing nitrogen (in legumes), and biosynthesizing plant hormones.^1,19,20^ Roots also play a key role in preventing lodging, which occurs when roots detach from the soil or stems bend due to external forces such as high wind. Stem bending damages the stem transport system, affecting the upward transport of water and nutrients from roots and the transport of photosynthetic products from leaves to ears, thus hampering seed setting and resulting in severe yield losses. Lodging also destroys leaf tissue, greatly reducing photosynthetic efficiency and thus yields, and hampers mechanical harvesting of crops.^21^ Lodging has become a major factor limiting maize yield.^22–24^

Plants typically have tap or fibrous root systems. The roots of soybean, canola, and other dicotyledonous plants are tap root systems, while wheat, rice, maize, and other monocotyledon plants have fibrous root systems. Tap root systems consist of a principal root and branch roots; the strongly developed principal root is thick and long and has geotropism and deep rooting ability, while the branch roots generated from the principal root are relatively short and thin, growing around the principal root at an angle. By contrast, fibrous root systems develop a mass of whisker-shaped roots with a shallow distribution and a large range, along with adventitious roots growing from the stem base.^25^

The development of branch roots involves the establishment of founder cells, development of lateral root primordia, and outgrowth of the branch root.^26,27^ Branch roots mainly originate in specific sheath cells of the maternal root that are stimulated to dedifferentiate and reestablish cell division to form lateral root primordia. Lateral roots grow from the lateral root primordia and the cells of the growing point continue to divide, enlarge, and differentiate. Root tip cells secrete enzymes to partially dissolve cortical and epidermal cells of the parent root, allowing the lateral roots to pass through the parent root epidermis into the external environment. This complex and orderly process is repeated during subsequent secondary and tertiary lateral root development.^28–30^ Moreover, the growth and development of plant lateral roots is affected by internal factors, such as hormones, and external factors, such as light, water, and nutrients, which regulate lateral root development by influencing intrinsic factors.^31–33^

BRs as plant hormones that regulate plant height and promote root growth. Moreover, BRs can induce plant tolerance to a variety of biological and abiotic stresses, such as heavy metal stress, salt stress, temperature stress, drought stress, pathogen infection, and oxidative stress.^34^ Low nitrogen specifically up regulates the transcription level of BRs co-receptor BAK1 to activate BRs signal and stimulate root elongation. The role of BR signal in root elongation under low nitrogen condition was revealed. The BRs signaling kinase BSK3 allele provides a target for improving root growth of crops growing under limited nitrogen conditions.^35,36^ Given their vital function in plant growth, development, and reproduction, exogenous application of BRs can increase the yield of various plants and improve crop quality.^37^ BRs is also nontoxic and beneficial to human health, acting as a potent antioxidant and neuroprotective agent in Parkinson’s disease^38^ and may improve keratitis in mice.^39^ BRs can increase plant yield^40^ with transgenic rice possessing increased BR biosynthesis levels showing yield increases of 15–44% per plant; BR can also enhance plant stress tolerance and reduce pesticide use, thus decreasing the amounts of pesticide residues.^41–43^ In rice, data on genes controlling growth and development downstream of BRs signal transduction are mainly derived from microarray results.^44,45^ Expression of *OsBLE1* increased markedly after BRs treatment, and transferring antisense *OsBLE1* into rice inhibits plant growth, indicating that *OsBLE1* is involved in BRs-regulated plant growth and development.^45^Expression levels of *OsMPD1* decrease in response to BRs treatment, and transgenic plants lacking *OsMPD1*are hypersensitive to exogenous BRs, showing that *OsMPD1* plays a negative role in BRs signal transduction.^44^ *OsMADS22* and *OsMADS55* are homologs of *OsMPD1* and have also been shown to play a negative regulatory role in BRs signal transduction [Shinyoung 46].

In this study, we used a multi-disciplinary approach based on plant morphology, developmental biology, plant physiology, cell biology, and molecular biology techniques to clarify the regulatory effects of BRs on maize root growth and development and the mechanism by which BRs improves disease resistance to improve maize yield.

## Materials and methods

### Plant materials and treatments

Maize (*Zea mays*) cultivar ‘B-73’ seeds of the same size and plumpness were kept at 30°C for 1 day. Root length was measured at two developmental stages. At the first stage, seeds were soaked in distilled water containing different concentrations of 2,4-epibrassinolide and placed on filter paper; root length was measured after germination at 28°C for 36 h. At the second stage, roots from seeds treated with 0, 0.5, 1, 1.5, or 2 mg/L eBL (an active by-product of BRs biosynthesis, which has the ability to stimulate different plant metabolic processes) were measured after planting seeds in soil for 15 days. Roots of maize grown on half-strength Murashige and Skoog (1/2 MS) medium containing 0 or 1 mg/L eBL for 3 days were observed by paraffin sectioning. When maize (Zhengdan 958) seedlings grew to the big bell mouth stage (between jointing and tasseling), 0 or 1 mg/L eBL was sprayed on the aboveground parts, and physiological indexes were measured after 1 week. After 4 weeks, the aerial root growth of maize was observed.

### *Fusarium* infection treatment

*Fusarium graminearum* and *Fusarium proliferatum* cultures showing good growth covering the entire surface of the medium were used for fungal inoculation tests. Suspensions of 10^6^ spores/mL were prepared. *F.proliferatum* was sprayed onto the aboveground parts of maize plants. *F.graminearum* spores were injected into maize stems at the big bell stage using a syringe. After inoculation, corn seedlings were watered as usual, and samples were taken 7 days or 4 weeks later to observe fungal infection.

### Proline content measurement

Fresh leaves (0.5 g) were harvested in a test tube, and 5 mL of 3% sulfosalicylic acid was added. After sealing the test tube with a glass ball, the sample was placed in boiling water for 10 min (shaking constantly to sink the crushed leaves). After cooling to room temperature, 2 mL of supernatant was mixed with 2 mL of glacial acetic acid and 3 mL of chromogenic solution in a fresh test tube (control tube: 2 mL of glacial acetic acid, 3 mL of chromogenic solution, and 2 mL of water) and placed in boiling water for 40 min. Toluene solution (5 mL) was added to each cooled test tube and shaken vigorously to extract the red substance. After standing for 10 min (to allow layers to form), the toluene layer was collected and its optical density (OD) was measured at 520 nm. The content of proline in the sample was calculated according to the formula y = (C* V)/(a * W), where C = 38.869 * OD_520_ + 0.0839;V = total volume of extract (mL); a = volume of toluene layer absorbed during determination (mL); and W = sample weight (g).

### Soluble sugar content measurement

Fresh corn leaves (0.3 g) were harvested into a test tube and mixed thoroughly with 9 mL of water, and the tube was placed in boiling water for 20 min. After cooling, the supernatant was poured off and water was added to the pellet to a final volume of 1 mL.Anthrone reagent (5 mL) was added, and the tube was placed in boiling water for 10 min. After cooling to room temperature, the OD was measured at 600 nm. Soluble sugar content (mg/g) = (C × V × n)/(v × w × 1000), where C = 2.4525 * OD_600_–0.0036 (C obtained using a standard curve (mg)); V = total volume of sample extract (mL); v = amount of sample added after sample extract (mL); w = fresh weight of sample (g); and n = dilution factor of the sample.

### Detection of superoxide dismutase (SOD) activity

A 50-μL aliquot of enzyme extract (5 mL phosphate buffer, pH7.8, 5 mL 0.2 M KNO_3_) plus 50 μL riboflavin was added to 4 mL of reaction solution (2 mL phosphate buffer, pH7.8, 0.5 mL 100 mM methionine, 1 mL 300 μM NBT, and 0.5 mL 0.8 mM EDTA) in a test tube. Two treatments contained no enzyme solution (replaced with pH7.8 phosphate buffer). Tubes were immediately placed under a 4000 LX fluorescent lamp for light reduction reaction. After 15 min, the reaction was stopped by shading with black paper. Phosphoric acid buffer was used as a zero point, and sample color was compared by measuring OD at 560 nm. SOD activity was calculated according to the formula (control OD_560_–sample OD_560_) *dilution factor/ (control OD_560_*0.5*w*sample dosage during determination), where w = fresh weight of sample (g).

### Malondialdehyde (MDA) content measurement

Thiobarbituric acid (TBA; 0.6%) was prepared by dissolving 0.6 g TBA in a small amount of 1 M NaOH by heating and adjusting the volume to 100 mL using 10% trichloroacetic acid (TCA). Enzyme extract (1 mL) and 2 mL of 0.6% TBA were added to a test tube, which was sealed and placed in a boiling water bath for 15 min. The sample was then allowed to stand before centrifuging at 12,000 *g* for 10 min. The OD of the supernatant was measured at 600, 532, and 450 nm. MDA content was determined using the following formula: MDA (μmol/g fresh weight) = (6.45×(OD_532_–OD_600_)–0.56× OD_450_) ×volume of extract (mL)/fresh weight of plant tissue (g).

### Detection of peroxidase (POD) activity

A 20 μL aliquot of enzyme extract (5 mL phosphate buffer, 5 mL 0.2 M KNO_3_) was mixed thoroughly with 3 mL of reaction solution (2.91 mL of phosphoric acid buffer, pH 7.0, 0.05 mL of guaiacol, 0.02 mL of 40 mM H_2_O_2_, 0.02 mL of enzyme solution) in a small tube and placed in a constant temperature water bath at 34°C for 3 min. Subsequently, 20 μL of 20% trichloroacetic acid was added to stop enzyme activity. Optical density was measured at 470 nm using pH7.0 phosphoric acid buffer as a zero point (the reaction solution was precooled in advance). POD activity was calculated according to the formula (sample OD_470_*total volume of enzyme solution)/(leaf fresh weight (g)*volume of enzyme solution (mL)).

### Measurement of chlorophyll content

Fresh leaves (0.2 g) were harvested and mixed with small amounts of quartz sand and calcium carbonate powder and 3 mL of 80% acetone in a mortar. The homogenate was transferred into a 25 mL Brown volumetric flask, and 80% acetone was added until the total volume reached 25 mL. The sample was then mixed and centrifuged at 12,000 *g* for 10 min. Using 80% acetone reagent as a control, OD was measured at 663 and 645 nm. Chlorophyll content was calculated as chlorophyll concentration * extract volume * dilution factor/sample fresh weight (g).

### Measurement of other physiological indexes

Other physiological indexes were measured using commercially available kits (Suzhou COMIN, China) for detection of hydrogen peroxide (Cat#H_2_O_2_-2-Y); protein (Cat# BCAP-2-W), crude fat (Cat# TFA-1-Y), and starch (Cat#DF-2-Y) content in grains; and phosphoenolpyruvate carboxylase (PEPC)(Cat#PEPC-2-Y), sucrose synthase (SS)(Cat#SSS-1-Y), and sucrose phosphate synthase (SPS)(Cat#SPS-2-Y) content in leaves.

### Stems tiffness measurement

A plant stem strength tester (YYD-1B; Zhejiang Tuopu, China) was used to measure stem lodging resistance of corn following different treatments. The front-end accessories of the plant stem strength tester and half of the corn stem were fixed with a rubber strip. Corn stems were gently pushed at right angles to the ground. Pressure values were recorded when maize stalks tilted to an angle of 45° with the ground.

### Reverse-transcription quantitative PCR (RT-qPCR)

Total RNA was isolated using Trizol reagent and digested using DNaseI to remove DNA contamination. cDNA was synthesized using oligo dT as the primer. *TUBULIN* genes were used as internal controls. Primers used for RT-qPCR are listed in Table S1. The reaction mixture (20 μL) contained 10 μL of SYBR Green Realtime PCR Master Mix (DBI, Bioscience, Germany), 0.5 μM each of forward and reverse primers, and 1 μL of cDNA template (equivalent to 50 ng of total RNA). PCR was performed on an ABI 7500 system using a SYBR Premix Ex Taq (perfect real time) kit (TaKaRa Biomedicals, Dalian, China) under the following conditions: 94°C for 30s and 40 cycles of 94°C for 5s, 58°C for 15s, 72°C for 10s, and 72°C for 7 min for plate reading. Three biological replicates were performed. Expression levels were calculated according to the delta-delta CT method.

### Statistical analysis

In the experiments involving statistical analysis, 15 seedlings were measured in each treatment. Three biological replicates were set up and the average value was taken. Each experiment was repeated at least three times, and the significance was analyzed by t-test.

## Results

### BRs promotes maize primary root growth

**Figure 1.**
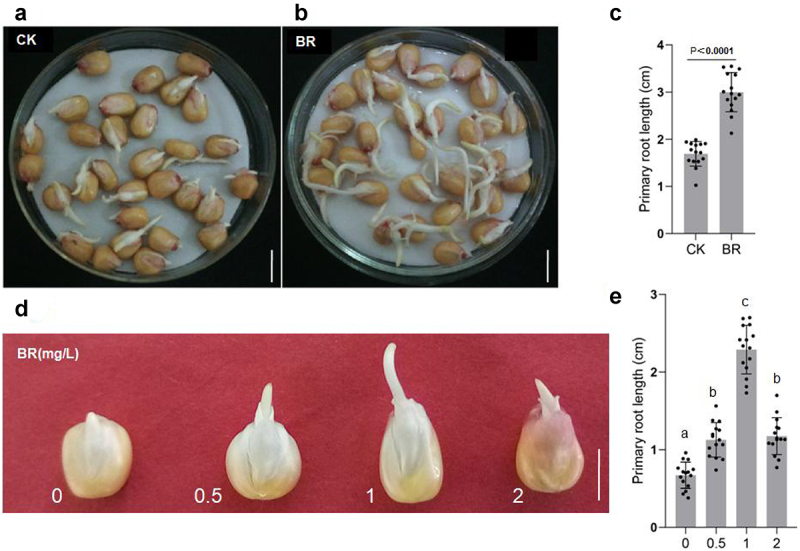
BR regulates the development of primary roots in maize. A, B, morphological observation of primary roots in maize grown under the indicated treatments. Control: 0 mg/L eBL; BR:1 mg/L eBL. Scale bars, 1 cm. C, statistics of primary root length of control(a) and BR(b) treatments. Data were analyzed from 15 seeds for each genotype from 3 experiments. Statistical significance was determined by student’s *t*-test (two-tailed) in (c). D, germination of maize seeds under 0, 0.5, 1, and 2 mg/L eBL treatments. Scale bars, 1 cm. E, statistics of primary root length after treatment with different concentrations of eBL(d). The letters indicate the significance groups at P < .05 (one-way ANOVA and Tukey test).

Roots absorb water and nutrients from the soil and anchor the plant to prevent lodging. Maize has a fibrous root system, in which the primary root from the embryo forms additional seminal roots; in maize, adventitious roots, called crown roots, also grow from the base of the stem.^25^ To observe the growth of maize primary roots, seeds of the same size and fullness were germinated at 28°C in distilled water containing no eBL or 1 mg/L eBL. After 2 days of BRs treatment, the primary roots of eBL-treated seeds were markedly longer than that in controls (Figure 1(a,c)), showing that BR could promote the growth of primary roots remarkably. To test the optimum concentration of BRs for regulating seed germination, we performed a germination experiment at 28°C for 36 h in distilled water containing 0, 0.5, 1, or 2 mg/L eBL. Primary roots were longest in distilled water containing 1 mg/L eBL, followed by the seeds germinated in 0.5 and 2 mg/L eBL distilled water, while seeds in distilled water without eBL showed the slowest germination (Figure 1(d,e)). Roots were significantly longer with 1 mg/L eBL treatment than with other treatments; however, the increasing trend of root length with eBL concentration was inhibited under 2 mg/L eBL treatment, demonstrating a typical concentration dependence of eBL treatment in which low concentration promotes and high concentration inhibits root growth. Therefore, these results indicate that BRs plays an important role in the elongation and development of maize primary roots. This is the same as the previous report (https://link.springer.com/article/10.1007/s10725-020-00626-z).

### BRs promotes secondary root growth

**Figure 2.**
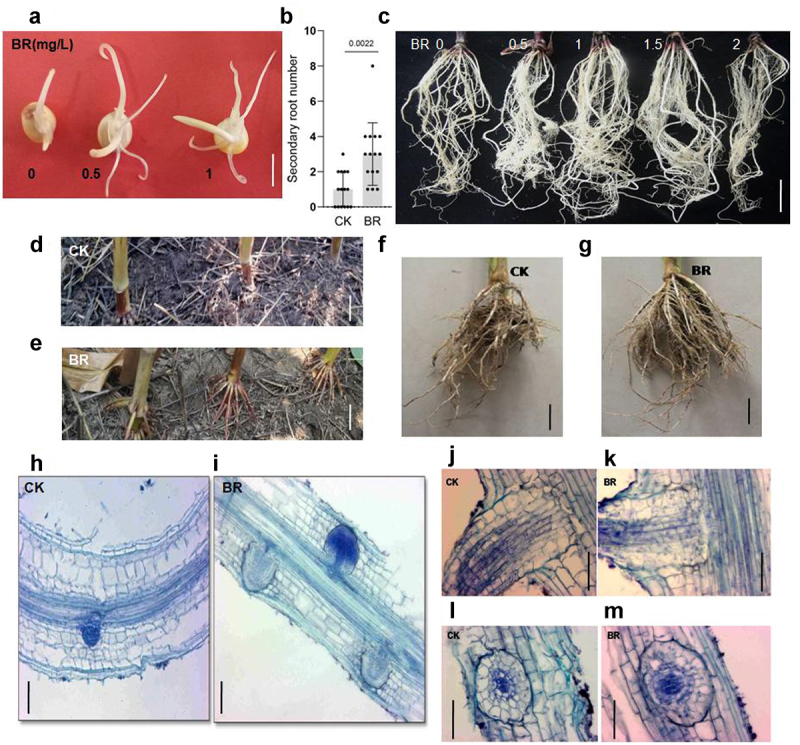
BR regulates the development of maize secondary and aerial roots. A, phenotype of maize secondary roots treated with different concentrations of eBL after 3 days. Scale bar: 1 cm. B, statistics of secondary root number treated with 0 and 1 mg/L eBL. Data were analyzed from 15 seeds for each genotype from 3 experiments. Statistical significance was determined by student’s *t*-test. P values are indicated. C. Phenotype of the maize secondary roots treated with 0, 0.5, 1, 1.5, and 2 mg/L eBL before planting in the soil after 15 days. Scale bar: 1 cm. D-G, phenotypes of aerial roots of the indicated treatment after 4 weeks of the big bell mouth stage. CK: 0 mg/L eBL (d, f); BR:1 mg/L eBL (e, g). Scale bars, 10 cm. H, I, phenotype of initial development of lateral roots in the indicated treatment after 3 days. CK: 0 mg/L eBL (h); BR:1 mg/L eBL (i). J-M, phenotype of lateral root transection and slitting in the indicated treatment after 3 days. CK: 0 mg/L eBL (j, l); BR:1 mg/L eBL (k, m).

Next, we tested the effect of BRs on secondary root length. When maize seeds were germinated for 3 days in eBL-containing distilled water, primary roots were obviously longer than those of control seeds. Furthermore, the number of secondary roots produced by eBL-treated seeds was also greater than that of the control (Figure 2(a,b)). In the process of maize root development, secondary roots, which are borne on all morphological roots, form from the embryo root. Hence, we planted maize seeds treated with 0, 0.5, 1, 1.5, or 2 mg/L eBL in soil and observed the growth of secondary roots after 15 days. The number of secondary roots treated with eBL were obviously greater than those in roots not treated with eBL. Lateral roots from seeds treated with 1 mg/L eBL displayed the highest density (Figure 2c), indicating that the development of maize secondary roots was promoted under BRs treatment.

Analysis of paraffin sections of maize primary roots grown on medium containing 0 or 1 mg/L eBL for 3 days showed that roots treated with no eBL produced only one lateral root, while BRs-treated roots had three lateral roots (Figure 2(h,i)), demonstrating that BRs can promote the early development of lateral roots. Further analysis of longitudinal and transverse sections of lateral roots showed that BRs treatment did not change the internal structure of lateral roots (Figure 2(j,m)).The function of BRs in promoting lateral root growth occurred during different growth periods of maize (aboveground or underground). After spraying BRs on the aboveground parts of maize at the big bell mouth stage, the development of underground lateral roots was also significantly promoted (figure 2(f,g)).

The unique aerial roots of maize provide strong lodging resistance. During the late stage of maize growth, the primary and secondary roots degenerate, and nutrition and water absorption are mainly dependent on aerial roots.^47,48^ Most importantly, ensuring normal yield under flood and drought conditions is crucial. We sprayed 1 mg/L eBL onto the shoots of maize seedlings with eight leaves to observe the effects of BRs on the growth and development of aerial roots. Aerial roots were observed after 4 weeks. The aerial roots of untreated controls were just beginning to grow; however, the aerial roots of BRs-treated maize had obviously elongated, and aerial roots developed in the previous stem section (Figure 2(d,e)). These results showed that BRs promotes the development of aerial roots in maize.

These results demonstrate that BRs promotes the development of maize primary, secondary, and aerial roots, with 1 mg/L eBL as the optimum concentration for promoting growth.

### BRs regulates root development via auxin

**Figure 3.**
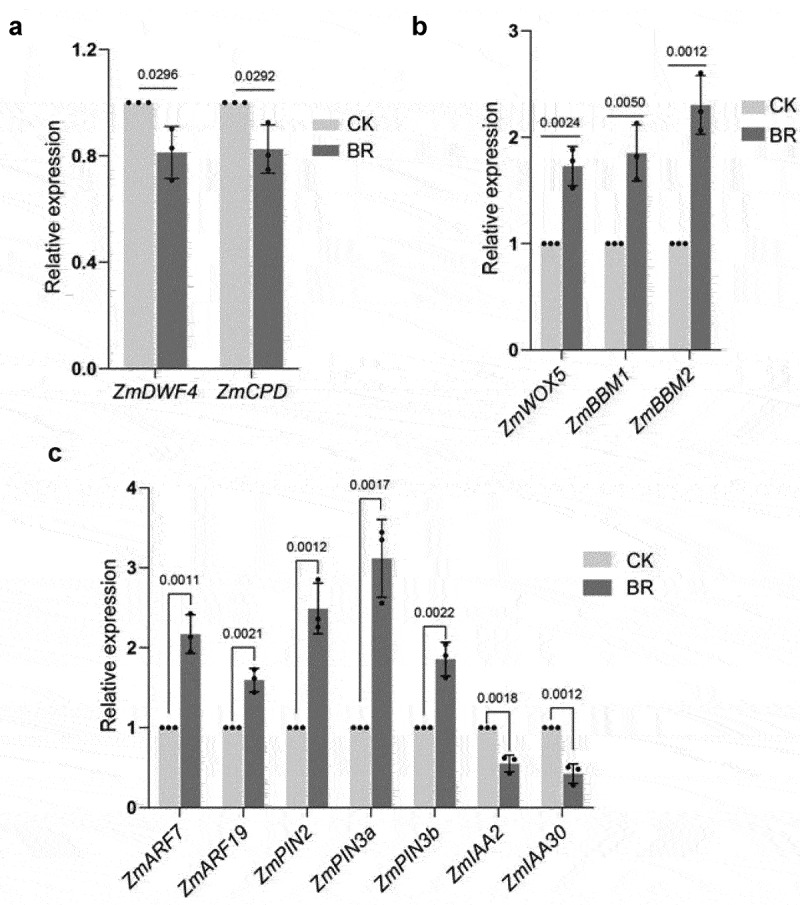
Expression analysis of key genes regulating root development. A, RT-qPCR of *ZmDWF4* and *ZmCPD*(a); *ZmWOX5, ZmBBM1*, and *ZmBBM2*(b); and *ZmPIN2, ZmPIN3a, ZmPIN3b, ZmIAA2, ZmIAA30,ZmARF7*, and *ZmARF19*(c)under the indicated treatments. CK: 0 mg/L eBL; BR:1 mg/L eBL. Gene expression in CK was set to 1. Data represent mean ± s.d. of three biological repeats. Statistical significance was determined by two-sided student’s t-test (A, B, C); NS, not significant; P values are indicated. *Tubulin* was used as an internal reference gene.

To further explore the role of BRs in maize root development, we analyzed the expression patterns of BR-related genes. The BRs biosynthesis genes *ZmDWF4*^49^ and *ZmCPD*^50–52^ were down-regulated by eBL treatment compared with untreated controls (Figure 3a), which was consistent with previous results in Arabidopsis (*Arabidopsis thaliana*), suggesting that this experimental treatment is effective.

*WOX5* is specifically expressed in the quiescent center (QC) cells in the Arabidopsis root meristem and maintains the activity of surrounding stem cells.^53,54^ The expression of *PLT1* and *PLT2* is closely related to auxin levels in the root meristem. *PLT* genes in Arabidopsis are necessary for the formation of the stem cell microenvironment in the root meristem. A PLT protein gradient controls stem cell division and cell differentiation.^55,56^We examined the expression of *WOX5, PLT1* and *PLT2* homologs *ZmWOX5, ZmBBM1*, and *ZmBBM2* in maize roots after BRs treatment and found that the expression of *ZmWOX5, ZmBBM1*, and *ZmBBM2* in BRs-treated roots was higher than that in untreated controls (Figure 3b).

Auxin participates in the ubiquitination and degradation of AUX/IAA proteins, releasing auxin response factors. Auxin response factors facilitate auxin-mediated regulation of gene expression.^57^ Auxin shows a gradient distribution in the root apical meristem, accumulating in the static center and stem cell area and gradually decreasing from the stem cell area to the root tip elongation area. We found that expression of the auxin transport genes *ZmPIN2, ZmPIN3a*, and *ZmPIN3b* was higher under BRs treatment than with no treatment (Figure 3c).*IAA14* and *IAA28* are inhibitors of lateral root primordium initiation,^58–61^ and their candidate maize homologs, *ZmIAA2* and *ZmIAA30*,^62^ were down-regulated after BRs treatment(Figure 3c). By contrast, some positive regulatory factors promoting the initiation of lateral root primordia, such as *ZmARF7* and *ZmARF19*,^63–66^ were up-regulated after BRs treatment (Figure 3c), suggesting that BRs regulates root growth and development through auxin.

### BRs enhances the stress tolerance of maize

**Figure 4.**
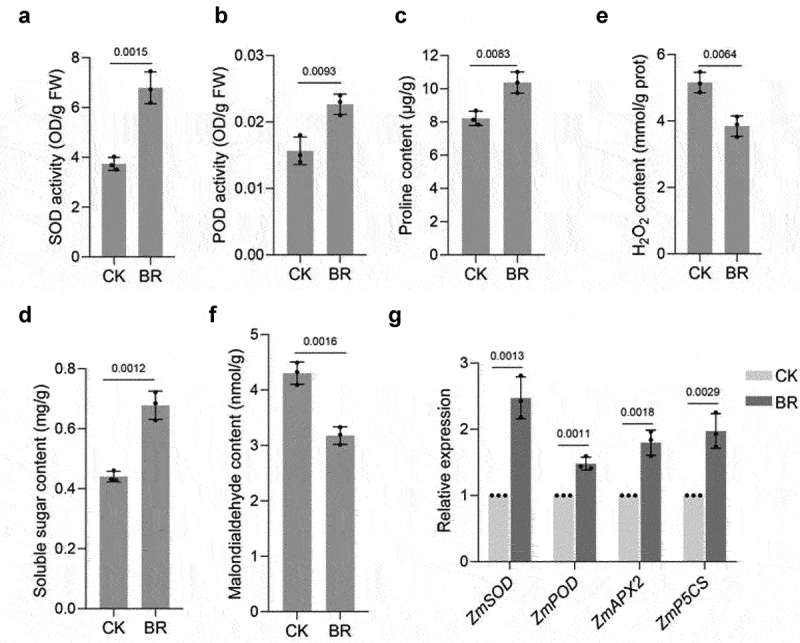
BR regulates physiological and biochemical indexes of maize. SOD(a), POD(b), proline(c), soluble sugar(d), H_2_O_2_(e), and MDA(f) content under the indicated treatments. CK: 0 mg/L eBL; BR:1 mg/L eBL. G, RT-qPCR of *ZmSOD, ZmPOD, ZmAPX2*, and *ZmP5CS* under the indicated treatments. CK: 0 mg/L eBL; BR:1 mg/L eBL. Gene expression in CK was set to 1. Data represent mean ± s.d. of three biological repeats. Statistical significance was determined by two-sided Student’s t-test (a-g); NS, not significant; P values are indicated. *Tubulin* was used as an internal reference gene.

We detected the SOD, POD, soluble sugar, proline, H_2_O_2_, and MDA contents in BRs-treated maize leaves at the big bell stage. SOD, POD, soluble sugar and proline contents in the leaves of maize treated with BRs were higher than those in the control (Figure 4(a,d)). Moreover, the H_2_O_2_ and MDA contents of BRs-treated leaves were lower than those of untreated controls (Figure 4(e,f)).At the same time, we examined the expression of *ZmSOD, ZmPOD, ZmAPX2*, and *ZmP5CS* after BRs treatment. The expression levels of *ZmSOD, ZmPOD, ZmAPX2*, and *ZmP5CS*were increased compared with those of the control (Figure 4g), suggesting that BRs treatment can enhance the stress tolerance of maize.

### BRs improves the resistance of maize to *Fusarium* infection

**Figure 5.**
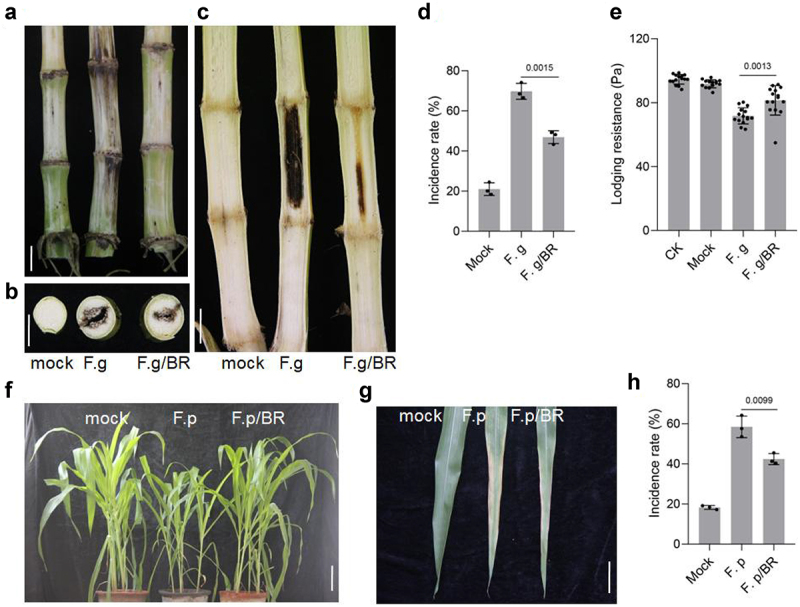
BR improved the resistance of maize. A-C, Phenotype of maize stalk under the indicated treatment(B, transaction; C, longitudinal section). Phenotype of maize seedling(f) and leaf(g) under the indicated treatment. Scale bars, 1 cm. The incidence rate(d,h) and lodging resistance(e), Data were analyzed from 15 seeds for each genotype from 3 experiments. Data represent mean ± s.d. of three biological repeats. Statistical significance was determined by two-sided Student’s t-test (D, E, H); NS, not significant; P values are indicated. CK: 0 mg/L eBL; BR:1 mg/L eBL.

*Fusarium graminearum* is one of the main pathogenic fungi causing corn stem rot.^67^ BRs can improve the ability of plants to resist a variety of stresses.^68^ To determine whether BRs treatment can improve the resistance of maize to *F.graminearum*, we inoculated maize treated with BRs and untreated maize with *F.graminearum. F.graminearum* spores were injected into maize stems at the big bell stage using a syringe. After inoculation, and samples were taken 4 weeks later to observe fungal infection. Treated maize stalks showed no obvious differences from the outside. When cut open, however, we found that the untreated maize stalks inoculated with *F.graminearum* were blackened over a large area, while the BRs-treated stalks had only a small blackened area. The incidence rate of *Fusarium* infection in plants treated with BRs was significantly lower than that of untreated plants (Figure 5(a,d)). The lodging resistance of untreated plants inoculated with *F.graminearum* was lower than that of BRs-treated plants. BRs treatment could therefore improve the lodging resistance of plants after *Fusarium* inoculation (Figure 5e).

*Fusarium proliferatum* is one of the main pathogens causing corn sheath rot.^69^ To determine whether BRs can prevent and control *F.proliferatum*, we inoculated BR-treated and untreated maize with *F.proliferatum. F.proliferatum* was sprayed onto the aboveground parts of maize plants, and samples were taken 7 days later to observe fungal infection. Plants infected with *F.proliferatum* were weaker, with reduced plant height, compared with control plants. The leaves showed large yellow patches. However, after BRs treatment, the incidence of *F.proliferatum* was low and plant height was similar to that of uninfected plants. Damage to the blade was less severe and plant growth was better than that in untreated plants inoculated with *F.proliferatum* (figure 5(f,h)).This suggested that the disease resistance of maize was weakened after being infected by *Fusarium*, while BRs improved the disease resistance of maize.

### BRs improves the photosynthetic performance of maize

**Figure 6.**
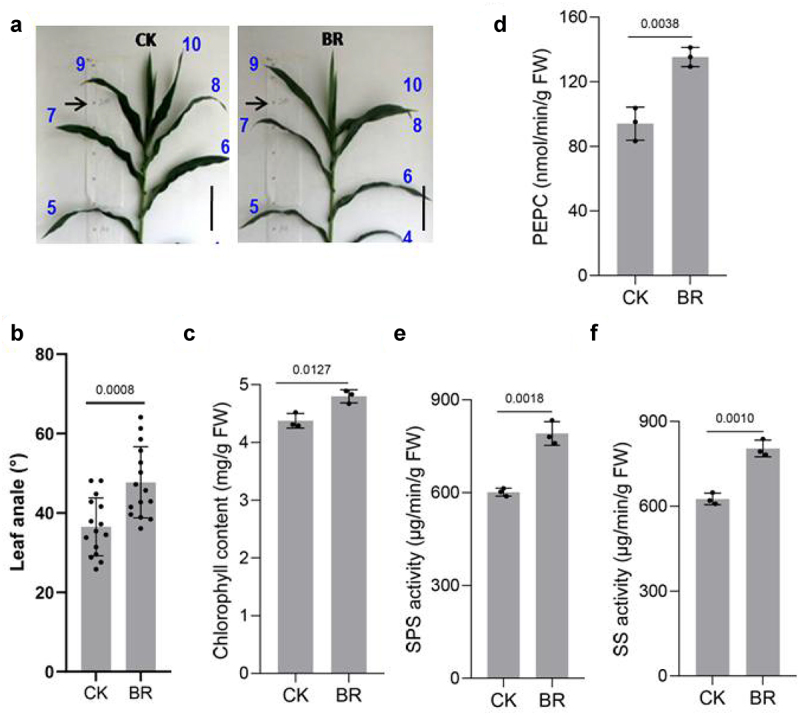
BR improves the photosynthetic performance of maize. Phenotype of maize leaf angle under CK and eBL treatments(a). Scale bars, 10 cm. And statistical data in leaf angle(b). Chlorophyll(c), PEPC(d), SPS(e), and SS(f) contents. Data represent mean ± s.d. of three biological repeats. Statistical significance was determined by two-sided Student’s t-test (d-f); NS, not significant; P values are indicated. CK: 0 mg/L eBL; BR:1 mg/L eBL.

To have practical significance, the ideal maize plant must have architecture suited to withstand close planting and possess lodging resistance. Our results showed that BRs promoted an increase in the leaf angle of maize (Figure 6(a,b)). Phosphoenolpyruvate carboxylase (PEPC) improves CO_2_ utilization and accelerates photosynthesis,^70–72^ while sucrose phosphate synthase (SPS) is the main enzyme for sucrose production, which is beneficial for storage of organic matter. SPS activity is positively correlated with photosynthetic rate.^73–75^ Sucrose synthase (SS) catalyzes free fructose and glucose in plants to produce sucrose.^76^ The activities of SPS and SS are positively correlated with grain filling, total starch, and amylopect in accumulation rate.^77^ We measured chlorophyll content^78^ and PEPC, SS, and SPS activities after spraying maize seedlings with BRs(Figure 6(c,f)). Chlorophyll content was slightly higher in BRs-treated plants than in non-BRs-treated plants. The activities of PEPC, SS, and SPS in plants sprayed with BR were significantly higher than those in control plants, indicating that BRs accelerated the photosynthetic rate of maize and the accumulation of organic matter, which promoted grain filling and played an important role in improving the yield and quality of maize.

### BRs increases the yield of maize

**Figure 7.**
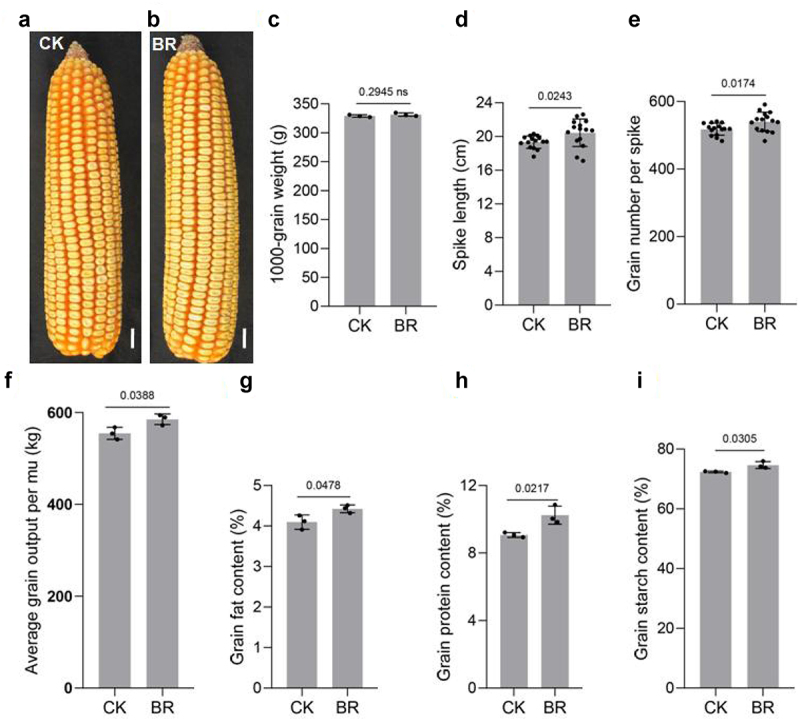
BR increases the yield of maize. Phenotype of maize spike length under CK(a) and eBL(b) treatments. Scale bars, 1 cm. The 1000-grain weight(c), spike length(d), grains per spike(e), and the yield per mu(f). Protein(h), fat(g), and starch(i) contents. Data were analyzed from 15 seeds for each genotype from 3 experiments(d, e). Data represent mean ± s.d. of three biological repeats. Statistical significance was determined by two-sided Student’s t-test (c-i); NS, not significant; P values are indicated. CK: 0 mg/L eBL; BR:1 mg/L eBL.

The value of crops depends on yield and quality.^79,80^ When maize seedlings grew to the big bell mouth stage (between jointing and tasseling), 0 or 1 mg/L eBL was sprayed on the aboveground parts, wait for the grain to mature. We therefore measured the yield of maize treated with BRs. The 1000-grain weight changed little after BRs treatment, but the spike length, number of grains per spike, and yield per mu all increased (Figure 7(a,f)). BRs treatment also increased the contents of protein, fat, and starch in grains (Figure 7(g,i)). This suggested that BR functions in improving grain yield and quality of maize.

## Discussion

Plant hormone treatment is an environmentally friendly, efficient method that is widely used to produce strong, stress-tolerant plants. BRs is an important plant hormone that not only affects plant growth, flowering, and photosynthesis^81,82^ but also increases the adaptability of plants to various biotic and abiotic stresses.^83^ Exogenous 0.025 g/L eBL treatment reduces the production of ROS by activating ROS-scavenging enzymes, regulating the plant osmotic state, and improving photosynthesis and other leaf gas exchange properties, thus playing an important role in plant stress responses.^84^ BRs treatment significantly increased the activities of antioxidant enzymes and enhanced the heat tolerance of kiwifruit seedlings, with 0.3 mg/L eBL being the most effective treatment. Selecting the optimal hormone concentration for spraying is crucial for crop growth and development under stress.^84–86^

Various abiotic stresses seriously affect maize production. For example, high-temperature stress accelerates leaf senescence, decreases leaf photosynthetic capacity and dry matter accumulation after anthesis, and increases grain abortion, which leads to yield losses.^87–89^ High-temperature stress before the silking stage significantly decreased the ear diameter and length, grain number per row, and 1000-grain weight of maize varieties Zhengdan 958 and Xianyu 335; it also increased the length of the bald tips of ears and decreased the yields of both varieties. High-temperature stress at the silking stage and grain completion stage significantly decreases the number of grains per ear and grain weight, thereby affecting yield.^90^ The main reason for this decreased grain number per ear is that high temperature increases the rate of grain abortion. Photosynthesis is blocked and respiration is enhanced under high-temperature stress, resulting in the decreased transport of assimilates from photosynthetic products to grains, which affects their accumulation in grains and grain weight, ultimately decreasing grain yield.^91,92^

Grain quality in maize is determined by the proportion of starch, protein, and fat and its internal structure.^93^ High-temperature stress not only reduces the grain yield of maize, but it also affects the grain quality of waxy maize. High-temperature stress increases starch grain size and protein content and decreases the grain/alcohol ratio.^94,95^ High-temperature stress accelerates grain filling, the degradation of starch in the pericarp, starch enrichment in endosperm cells, and starch accumulation, but the duration of continuous grain filling is shortened and cell division is reduced, resulting in a significant decrease in starch content at maturity.^96^ High temperature at the grain filling stage decreases the photosynthetic rate, starch synthase activity, and starch content, resulting in starch loss and smaller granules. The decrease in total soluble sugar and starch contents in maize grains under high-temperature stress before and after anthesis is mainly due to the decreased activity of starch biosynthesis-related enzymes such as FBP, SPS, SS, and ADPG, which leads to the deterioration of maize quality.^97^

Exogenous hormones play important roles in alleviating the effects of high-temperature stress on starch quality. Exogenous application of plant hormones (BRs, methyl jasmonate, and so on) enhances photosynthesis in rice under high-temperature stress, promotes the transport of carbohydrates to young spikelets, increases the seed-setting rate and grain-filling rate of spikelets, and alleviates damage to grain formation caused by high temperature.^98,99^

Stem rot disease poses a great threat to maize and has become an important factor limiting maize production. Stem rot is a typical soil-borne disease caused by diverse pathogens and is difficult to control, as there is currently no ideal method for doing so.^100–102^ The breeding of resistant varieties, strengthening field cultivation management, the proper application of fertilizers, and the use of conventional chemicals are effective measures for limiting disease severity.

BRs regulates plant growth and development; therefore, studying the functions of BRs regulatory proteins is important for clarifying their mechanisms of action. To date, few studies have focused on the functions of BRs regulatory proteins, and the mechanism underlying the roles of BRs in regulating plant growth and development are not clear.^103–105^ There is an urgent need for research and development of regulators that increase yields, stress resistance, and lodging to help solve current food security problems, increase food production, decrease the need for chemical fertilizers and pesticides for food production, improve crop stress resistance, and reduce the loss of production caused by natural disasters.

**Figure 8.**
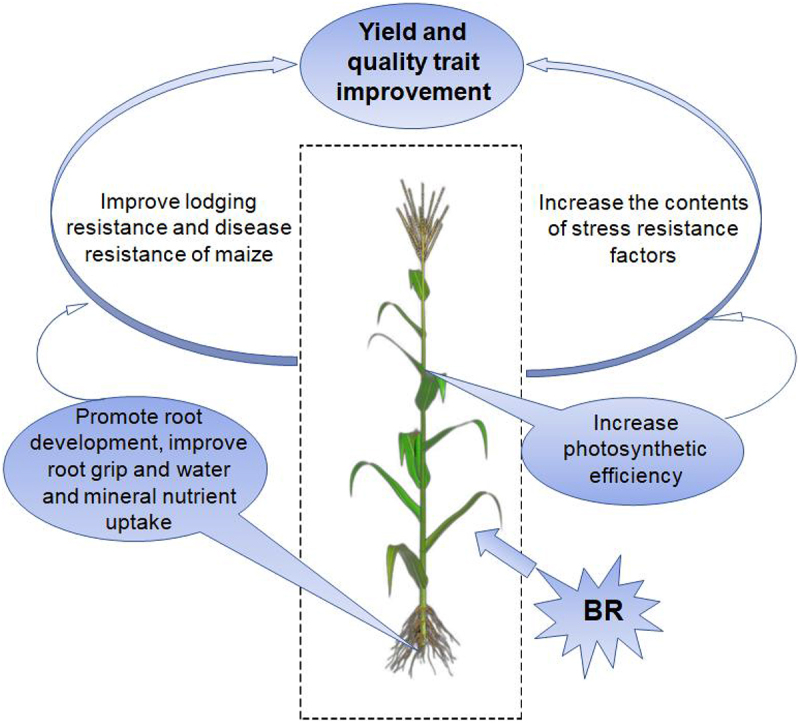
Model of increasing maize yield by BR.

In this study, BRs treatment promoted the development of primary, lateral, and aerial roots and increased the root area for the absorption of nutrients and water in maize. In addition, BRs treatment enhanced the nitrogen fixation ability of the plant, improved lodging resistance, reduced MDA and hydrogen peroxide contents, and increased the contents of substances that enhance tolerance to various abiotic stresses, such as proline, SOD, and POD. Furthermore, BRs promoted plant disease resistance and ultimately increased the yield of maize (Figure 8). These results highlight the potential value of BRs treatment for enhancing agricultural production.

## Supplementary Material

Supplemental Material
